# Daily Vaginal Application of Dienogest (Visanne©) for 3 Months in Symptomatic Deeply Infiltrating Rectovaginal Endometriosis: A Possible New Treatment Approach?

**DOI:** 10.1155/2018/8175870

**Published:** 2018-05-10

**Authors:** Andreas D. Ebert

**Affiliations:** Praxis für Frauengesundheit, Gynäkologie und Geburtshilfe, Nürnberger Strasse 67, 10787 Berlin, Germany

## Abstract

A 27-year-old patient suffering from deeply infiltrating rectovaginal endometriosis was treated with 2 mg/day dienogest vaginally for 3 months. The therapy was tolerated very well. The patient reported less side effects compared to the oral use of dienogest. After 3 months of dienogest treatment, the rectovaginal gynecological examination identified the visible vaginal part of endometriosis in remission. The firm endometriosis node approximately 3 cm in size and approximately 10 cm ab ano was still palpable, but it was much less painful. The laboratory values for luteinizing hormone (LH) and follicle-stimulating hormone (FSH) were unremarkable, with an LH/FSH quotient of 0.7 during dienogest treatment, while 17-*β* estradiol and progesterone were suppressed. At palpation and vaginal ultrasonography, there was no change in the findings before and after 3 months of dienogest treatment, but the patient was now de facto asymptomatic. To the best of our knowledge, this is the first report of a vaginal dienogest treatment in symptomatic deeply infiltrating rectovaginal endometriosis. Vaginal administration of dienogest should receive further investigation in pharmacokinetic and clinical studies.

## 1. Introduction

Symptomatic deeply infiltrating rectovaginal endometriosis with bowel involvement is a diagnostic and therapeutic challenge [1–5].

*Oral* administration of dienogest (Visanne©) is currently approved in Germany for the treatment of endometriosis alongside subcutaneous application of leuprorelin acetate (Enantone©, Trenantone©). Due to its lower costs and narrower range of side effects, dienogest is currently the drug treatment of choice in comparison with gonadotropin-releasing hormone (GnRH) analogues [6]. In terms of their effects and side effects, the two treatment approaches are equivalent [7–10]. Research studies to date have been carried out with oral administration of dienogest 2 mg/d [11–14]. To the best of our knowledge, there have as yet been no reports on trials of vaginal treatment with 2 mg/d dienogest in patients with symptomatic deeply infiltrating endometriosis.

## 2. Case Presentation

**Figure 1:**
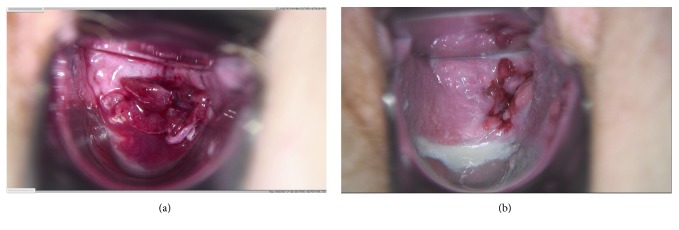
(a) Fresh, vulnerable, histologically confirmed rectovaginal endometriosis in the posterior fornix (Medivan video colposcope). The visual analogue score for the examination (VAS_examination_) before dienogest therapy was 9. (b) Remission of the same endometriotic lesion in the posterior fornix 3 months later after daily vaginal dienogest application. VAS_examination_ score was now 4.

**Figure 2:**
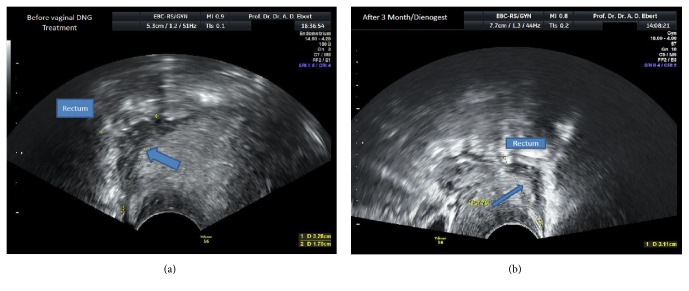
(a) Vaginal ultrasound appearance of the rectovaginal bowel involvement before the start of treatment in the symptomatic patient. (b) No change in the vaginal ultrasound findings after 3 months of vaginal dienogest administration (2 mg/d) in the patient who was now de facto asymptomatic.

A 27-year-old woman (gravida I, para I; menarche at age 13) presented on 1 October 2017 due to secondary dysmenorrhea that she was suffering from since the age of 25, dyspareunia with back pain, constipation with perimenstrual tympanites, and contact bleeding in a case of known rectovaginal endometriosis. Her menstrual cycle was regular (27/4). Her visual analogue score for dysmenorrhea (VAS_dysmenorrhea_) was 8. A rectovaginal gynecological examination revealed fresh endometriosis in the posterior fornix, with slight bleeding (Figure 1(a)). The vaginal part of the cervix was unremarkable on colposcopy, cytology, and smear testing. At approximately 10 cm ab ano, palpation identified a typical firm, painful node with a diameter of approximately 3 cm, and poorly displaceable bowel mucosa, which was also clearly visible on vaginal ultrasonography (Figure 2(a)). The bilateral renal ultrasound findings were unremarkable. It was known from the patient's history that in February 2015 she had undergone a laparoscopy at a different hospital due to symptoms and wanting to have a baby; histology had confirmed deeply infiltrating endometriosis without atypia in the posterior fornix and pouch of Douglas. Adequate removal of the endometriosis was not carried out. The tubes were bilaterally patent. Postoperatively, the patient had taken dienogest (2 mg/d orally) up until August 2015, but she had very poor tolerance for it due to side effects (effluvium, blemished skin, sad mood). She became pregnant in October 2015 and delivered a boy at term in 2016 by emergency cesarean section due to premature placental detachment.

The endometriosis-related symptoms increased postpartum, with progressive deterioration in her quality of life. Despite this, the patient declined surgery due to fear of complications and for family and social reasons. She also declined endocrine treatment options (gonadotropin-releasing hormone antagonists or agonists, progestin-only pills, and oral contraceptives) due to the possible side effects. For this reason, the option of vaginal application of dienogest was discussed with the patient and implemented. The patient presented again after 3 months of vaginal dienogest treatment (2 mg/d vaginally). She reported minor vaginal spotting during the first 4 weeks of the treatment but had been amenorrhoeic for just under 8 weeks. She reported very good satisfaction with the treatment, after transient minimal side effects initially (slight skin blemishes, minimal discharge), and symptomatic freedom from the relevant endometriosis-related symptoms. Slight contact bleeding occurred only during sex. No vaginal infections had occurred during the treatment period. The rectovaginal gynecological examination identified the endometriosis in remission in the posterior fornix (Figure 1(b)). The firm endometriosis node approximately 3 cm in size and approximately 10 cm ab ano was still palpable, but it was much less painful (Figure 2(a)). Bilateral renal ultrasonography was unremarkable. The laboratory values for luteinizing hormone (LH; 5.07 U/L) and follicle-stimulating hormone (FSH; 7.29 U/L) were unremarkable, with an LH/FSH quotient of 0.7 during dienogest treatment, while 17-*β* estradiol (24.2 pg/mL) and progesterone (<0.05 ng/mL) were suppressed. At palpation and vaginal ultrasonography, there was no change in the findings before and after 3 months of dienogest treatment, but the patient was now de facto asymptomatic. In addition, the vaginal part of endometriosis was clearly in remission as demonstrated by vaginal colposcopy (Figures 1(a) and 1(b)). At her express request, continuation of the vaginal dienogest treatment was agreed.

## 3. Discussion

In the present case, vaginal dienogest (2 mg/d) was administered for 3 months. During the treatment, remission occurred in the area of the vaginally visible part of the deeply infiltrating endometriosis (Figures 1(a) and 1(b)), while the rectal part showed no significant changes in size on palpation or ultrasonography (Figures 2(a) and 2(b)). Despite this, the patient became free of symptoms apart from contact bleeding—a major therapeutic success. The rectovaginal examination was also significantly less painful after 3 months of dienogest (VAS_examination_ 9 versus VAS_examination_ 4). These findings are in accordance with our own experience and experience reported by others: before the size of the node declines, symptoms initially improve [14]. Despite the higher local dosage of dienogest, a reduction in the size of rectovaginal endometriosis nodes can probably only be observed clinically later on, since rectovaginal endometriosis always represents as* adenomyofibromatous* lesion [15], treatment-related remission of which takes time [16]. Dienogest was effectively resorbed vaginally, leading to therapeutic amenorrhea and the corresponding hormone findings in the present patient.

In principle, vaginal application of dienogest may be able to circumvent the hepatic first-pass effect and achieve higher concentrations of the active agent at the site of effect (e.g., a rectovaginal endometriosis node) with lower side effects than with oral administration [17]. The present patient thus reported that she found the vaginal application of dienogest much more tolerable than oral intake. A reduction in the dosage would also be conceivable with local dienogest administration. Studies with danazol, anastrozole, and contraceptive rings have shown that there is a reduction in lower abdominal pain and particularly in dysmenorrhea. However, a reduction in the size of rectovaginal nodes measured using ultrasound has not been reported by all investigators [17].

With vaginal application of dienogest (or other steroids), higher hormone concentrations can be achieved at the rectovaginal endometriosis node than with oral, intramuscular, or transcutaneous administration [17]. A prerequisite for this is correct placement of the active agents in the upper third of the vagina. For patients with symptomatic rectovaginal endometriosis who decline surgery or other treatment options for various reasons, vaginal dienogest application may thus represent a new approach to treatment, with low side effects. As vaginal application is familiar with other drugs [17], vaginal administration of dienogest (as well as other hormones) should receive further investigation in pharmacokinetic and clinical studies.
